# The effect of H_2_O on the sulfation of Havelock limestone under oxy-fuel conditions in a thermogravimetric analyser

**DOI:** 10.3906/kim-2008-4

**Published:** 2021-04-28

**Authors:** Murat VAROL, Edward John ANTHONY, Arturo MACCHI

**Affiliations:** 1 Department of Environmental Engineering, Middle East Technical University, Ankara Turkey; 2 Department of Environmental Engineering, Akdeniz University, Antalya Turkey; 3 Natural Resources Canada, CanmetENERGY, Ottawa Canada; 4 Cranfield University, Cranfield, Bedfordshire United Kingdom; 5 Department of Chemical and Biological Engineering, University of Ottawa, Ottawa Canada

**Keywords:** Oxy-fuel combustion, sulfation, limestone, water vapor

## Abstract

A gas mixture representing oxy-fuel combustion conditions was employed in a thermogravimetric analyser to determine the effect of water vapor and SO_2_ concentration on limestone sulfation kinetics over the temperature range of 800 to 920 °C. Here, experiments used small samples of particles (4 mg), with small particle sizes (d_p_ < 38 µm) and large gas flow rates (120 mL/min@NTP) in order to minimize mass transfer interferences. The gas mixture contained 5000 ppm_v_ SO_2_, 2% O_2_, and the H_2_O content was changed from 0% to 25% with the balance CO_2_. When water vapor was added to the gas mixture at lower temperatures (800–870 °C), the limestone SO_2_ capture efficiency increased. However, as the temperature became higher, the enhancement in total conversion values decreased. As expected, Havelock limestone at higher temperatures (890 °C, 920 °C, and 950 °C) experienced indirect sulfation and reacted at a faster rate than for lower temperatures (800–870 °C) for direct sulfation over the first five minutes of reaction time. However, the total conversion of Havelock limestone for direct sulfation was generally greater than for indirect sulfation.

## 1. Introduction

Oxy-fuel combustion is one of the methods available for carbon capture and storage (CCS). While the primary purpose of oxy-fuel combustion is to produce a nearly pure CO_2_ stream to reduce the cost of CO_2_ capture, the technology also offers the possibility of building smaller plants with a possible saving of around 30% of capital cost [1]. Oxy-fuel combustion also offers a number of additional advantages including the fact that the NOx emissions are smaller for a given thermal input of fuel. Due to its advantages, oxy-fuel combustion is one of the most promising technology for CCS [1–6], moreover the possibility of carrying out cocombustion with biomass, may also offer the possibility of negative CO_2_ emissions via the so called bio-energy with carbon capture and storage (BECCS) approach [7,8]. Biomass is assumed to be “CO_2_ neutral” because it uses CO_2_ in the atmosphere as a carbon source during its growth and if CO_2_ released by the combustion of biomass is captured, then the CO_2_ is permanently removed from the atmosphere and hence BECCS technology can achieve negative CO_2_ emissions [9].

Limestone is used in order to minimize sulfur emissions from both air and oxy-fuel fired circulating fluidized bed (CFB) combustors. In air firing, sulfur removal by limestone occurs in two sequential steps: calcination (R1), sulfation (R2) [10]. For sulfur capture, under air firing it is normally assumed that limestone first calcines and then the resulting CaO reacts with SO_2_ to form CaSO_4_ (sulfation). Collectively, these two reactions (R1 and R2) are known as indirect sulfation. Sulfation itself is characterized by two distinct reaction regimes: a first regime in which the reaction rate is controlled by chemical kinetics and intra-pore gas diffusion and a second one where the kinetic rate drops substantially as the reaction switches to a diffusion-limited process once a product layer (CaSO_4_) forms and covers the surfaces of larger pores, and plugs smaller pores due to the higher molar volume of CaSO_4_. The sulfur capture efficiency for indirect sulfation is limited because of pore blockage [10–14].

CaCO_3_ → CaO + CO_2_ (R1)

CaO + ½ O_2_ + SO_2_ → CaSO_4_ (R2)

CaCO_3_ + ½ O_2_ + SO_2_ → CaSO_4_ + CO_2_ (R3)

The partial pressure of CO_2_ is an important factor affecting the calcination of limestone. If the partial pressure of CO_2_ in the combustion medium is high, this can prevent calcination. High CO_2_ concentration in the flue gas for oxy-fuel combustion reduces the formation of CaO by reaction (R1) if the temperature in the combustor is less than the decomposition temperature of CaCO_3_ [14-16]. The decomposition temperature of CaCO_3_ (T, in K) can be calculated according to the formula (Eq.1) below [16]. Here, P_e_ in the equation is the partial pressure of CO_2_ in combustion medium in units of kPa. Approximately 80% of the gas mixture in the combustor during typical oxy-fuel combustion is CO_2_ (Pe is around 81 kPa). Under this condition, the decomposition temperature of CaCO_3_ is about 880 °C. Below that temperature, calcination of limestone does not occur in oxy-fuel conditions. Circulating fluidized bed (CFB) combustors are generally operated around 850 °C; thus, if a typical CFB is operated at 850 °C for oxy-fuel conditions, limestone will not calcine. Therefore, sulfation will proceed through the direct reaction of CaCO_3_ with SO_2_ (R3), which is known as direct sulfation [17].

Log P_e_ = 9.08 – 8308/T (Eq.1)

For coal-fired CFB combustors, the flue gases usually consist of 15% CO_2_, 3%–5% O_2_, 5%–15% H_2_O, and small amounts of SO_x_, NO_x_, with the balance N_2_. CFB technology is also often used to burn low quality fuels (high ash and high moisture). Under those conditions, moisture content in the flue gas may be even higher [10]. In the case of oxy-fuel combustion, the water vapor content also becomes extremely high in the combustor if the water vapor in the recycled flue gas is not condensed. If fuels with high H (e.g., natural gas) are burned, the water vapor content in the flue gas may even reach up to 70% for the oxy-fuel combustion in an O_2_/H_2_O atmosphere [18-19]. Although there are some studies on sulfation of limestone under high water vapor content, disagreements regarding the mechanism responsible require further investigation [20-22]. Here, our aim is to study limestone in atmospheres containing different percentages of CO_2_, O_2_, SO_2_, and H_2_O using a thermo-gravimetric analyzer (TGA) to help elucidate the sulfation mechanism.

Although the sulfation of limestone is well studied, the effect of water vapor has not yet been fully elucidated. Despite a few studies stating that sulfation rate of CaO decreased with an increase in water vapor [23], most studies suggest the opposite, namely a positive effect of water vapor on sulfation [10, 11, 17, 20, 22, 24, 25]. Some studies suggest that the transient formation of Ca(OH)_2_ promotes the sulfation reaction of the limestone [10, 17], but other studies suggest that enhanced sulfation is only effective in the diffusion-controlled stage [11, 20, 24]. Therefore, more work needs to be done on the subject to resolve this issue. Moreover, most past experiments were only conducted over a fairly narrow temperature range of 800–850 °C and the maximum percentage of water vapor was 15% [10, 11, 17, 21, 22]. This study explores the effect of high amounts of water vapor (25%) and a wider temperature range (800–950 °C). This study is expected to contribute to the literature in terms of examining the effect of water vapor on the conversion of limestone under oxy-fuel combustion conditions at high temperatures (890–950 °C).

## 2. Materials and methods

Gas mixtures representing typical oxy-fuel combustion conditions were introduced into the thermogravimetry a thermo-gravimetric analyzer in order to determine the effect of the amount of water vapor and SO_2_ concentration in the gas mixture on limestone sulfation. Havelock limestone was used in the tests, and its composition is given in Table 1. In order to determine the effect of the water vapor on the indirect and direct sulfation of Havelock limestone, 4 mg samples of Havelock limestone were exposed to gas mixture at six different temperatures, 800 °C, 830 °C, 870 °C, 890 °C, 920 °C, and 950 °C, respectively. The gas mixture was composed of 5000 ppm_v_ SO_2_, 2% O_2_, and H_2_O changing from 0% to 25%. The total flowrate of the gas mixture was 120 mL/min@NTP (T = 20 °C, p=101.3 kPa) and the balance of the gas mixture was CO_2_. For the reaction kinetic studies, the smallest size particles (<38 µg), the greatest gas flow rates (120 mL/min) and small quantity of particles (4 mg) were used to minimize transport interferences. The conditions for the TGA tests are given in Table 2 below.

**Table 1:** Analysis of Havelock limestone (wt%).SiO_2_2.00MgO1.91Al2O30.44SO3<0.10Fe2O31.47Na2O<0.20TiO_2_<0.03K2O0.03P2O5<0.03Loss of fusion45.21CaO48.81Sum99.99

SiO_2_	2.00	MgO	1.91
Al2O3	0.44	SO3	<0.10
Fe2O3	1.47	Na2O	<0.20
TiO_2_	<0.03	K2O	0.03
P2O5	<0.03	Loss of fusion	45.21
CaO	48.81	Sum	99.99

**Table 2:** List of TGA tests.

#	H_2_O content in the gas mixture (%)*	temperature (°C)	#	H_2_O content in the gas mixture (%)*	temperature (°C)
1	0.0	800	14	0.0	890
2	10.0	800	15	10.0	890
3	15.0	800	16	15.0	890
4	20.0	800	17	20.0	890
5	25.0	800	18	25.0	890
6	0.0	835	19	0.0	920
7	10.0	835	20	10.0	920
8	15.0	835	21	15.0	920
9	25.0	835	22	20.0	920
10	0.0	870	23	25.0	920
11	10.0	870	24	0.0	950
12	15.0	870	25	10.0	950
13	20.0	870	26	15.0	950
			27	20.0	950
			28	25.0	950

All experiments followed the same procedure. A known amount of sample (about 4 mg) was loaded into the TGA in a sample pan and then heated from room temperature to the final temperature at a rate of 40 °C/min under an O_2_/CO_2_ environment. Prior to the injection of SO_2_ and steam mixture to the TGA, the O_2_/CO_2_ ratio was adjusted according to the water vapor content in the gas mixture. Here, it was 0.021, 0.023, 0.024, 0.026, and 0.028 for the tests where the gas mixture containing 0%, 10%, 15%, 20%, and 25% H_2_O respectively. Once the preset temperature was reached, the weight of the limestone was measured and when it was constant for about 10 min, SO_2_ and steam mixture were simultaneously introduced to the TGA. After introducing the SO_2_ and steam mixture, the sulfation of limestone particles was determined from the weight increase. The flow rate was maintained at 120 mL/min throughout the test, and sample weights and the temperature were recorded continuously [10].

The decomposition temperature of CaCO_3_ during oxy-fuel combustion (80% CO_2_ by volume) is calculated to be 880 °C from Eq.1. It is assumed that at temperatures lower than that the decomposition temperature of CaCO_3_, limestone is not calcined during oxy-fuel combustion and that the direct reaction of CaCO_3_ with SO_2_ occurs according to the reaction (R3) [24]. For the tests conducted at these temperatures (800, 835 and 870 °C), the total conversion was calculated according to the formula (Eq.2) given below [24]. The total conversion for direct sulfation is defined as the molar ratio of CaSO_4_ formed after sulfation at any time t to the initial amount of CaCO_3_ in the limestone sample.

(Eq.2)Total conversion (%) (direct sulfation) = (m2-m1)m1*A*MWCaCO3MWCaSO4-MWCaCO3*100

m_1_: weight of the sample just before sulfation, g

m_2_: sample mass at time t, g

A: mass fraction of CaCO_3_ in the sample (assumed as 0.92), -

MWCaCO_3_: molecular weight of CaCO_3_, 100.09 g/mole

MWCaSO_4_: molecular weight of CaSO_4_, 136.14 g/mole

At temperatures higher than that the decomposition temperature of CaCO_3_, limestone decomposes to CaO during oxy-fuel combustion and then the CaO will react with SO_2_ according to reaction (R2). For the tests conducted at these temperatures (890, 920, and 950 °C), the total conversion was calculated according to the formula (Eq.3) given below [22]. Eq.2 can be used for indirect sulfation. In that case, the molecular weight of CaCO_3_ is replaced by the molecular weight of CaO.

(Eq.3)Total conversion (%) (indirect sulfation) = (m2-m1)m1*A*MWCaOMWCaSO4-MWCaO*100

MW_CaO_: molecular weight of CaO, 56.08 g/mole

## 3. Results and discussion

The total conversion of limestone and the sulfation time for the total conversion according to the TGA temperature and H_2_O/CO_2_ ratio is given in Table 3. The total conversion for each test was calculated according to the weight of the samples measured in TGA just before the sulfation and at the end of the sulfation time. The mass fraction of CaCO_3_ in the limestone sample (A in Eq.2 and Eq.3) was assumed as 0.92 for the conversion calculations but can vary considering the small size of the particles and the sample mass. Therefore, “~100” was used in Table 3 for the conversion values slightly greater than 100. For the tests where the conversion was over 100% (~100), the limestone sample in the TGA slightly exceeded the mass measured just before sulfation started. This means that CaCO_3_/CaO was completely converted to CaSO_4_ during sulfation process.

**Table 3:** TTotal conversion of Havelock limestone according to the TGA temperature and H_2_O/CO_2_ ratio.

TTGA (°C)	H_2_O (%)	H_2_O/CO_2_ (-)	Duration ofsulfation (min)	Totalconversion (%)	Conv. after 28 min ofsulfation (%)
800	0	0.00	30	60.6	59.0
800	10	0.11	35	80.9	72.4
800	15	0.18	35	68.8	62.7
800	20	0.26	40	87.2	72.9
800	25	0.34	39	89.9	79.3
835	0	0.00	34	84.7	79.5
835	10	0.11	41	98.3	84.9
835	15	0.18	36	89.0	81.5
835	25	0.34	79	52.2	38.5
870	0	0.00	67	~100.0	85.5
870	10	0.11	42	~100.0	98.7
870	15	0.18	37	95.4	85.4
870	20	0.26	41	~100.0	92.5
890	0	0.00	41	27.4	25.2
890	10	0.11	37	43.0	40.3
890	15	0.18	37	30.2	28.3
890	20	0.26	41	72.0	66.0
890	25	0.34	47	75.9	66.3
920	0	0.00	38	61.8	61.8
920	10	0.11	38	78.9	73.8
920	15	0.18	33	74.8	72.1
920	20	0.26	43	69.9	62.7
920	25	0.34	36	72.3	67.5
950	0	0.00	44	82.2	75.6
950	10	0.11	29	~100.0	~100.0
950	15	0.18	39	75.4	70.1
950	20	0.26	36	82.6	77.7
950	25	0.34	44	80.5	74.2

In order to compare the conversion over the same time period, the minimum sulfation time was determined. It was 28 min for the test without water vapor at 920 °C. That time period is taken as a reference for the comparison of conversion values. The conversion values after 28 min of sulfation for each test were calculated and provided in Table 3 (6th column). When the total conversion was compared to conversion after 28 min of sulfation with respect to the water vapor content and the temperature, it can be seen that they have the same trend.

**Figure 1:**
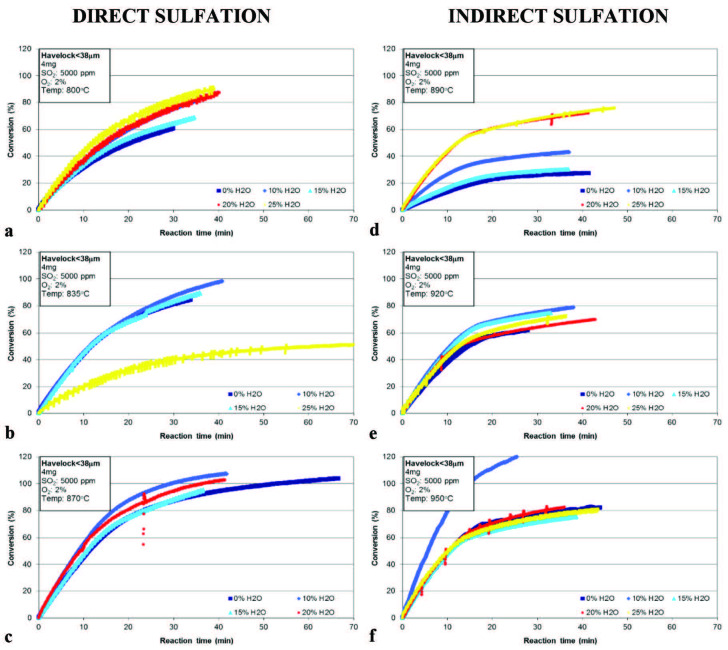
Effect of H_2_O on direct and indirect sulfation of Havelock limestone.

The results of the sulfation for Havelock limestone are given in Figure 1. From Figure 1a, it can be seen that the presence of the H_2_O in gas mixture at 800 °C improved the conversion of limestone during the sulfation process. While the conversion after 28 min of sulfation was 59% (Table 3) without H_2_O at 800 °C, 22.7%, 23.6%, and 34.4% of improvements were achieved by introducing 10%, 20% and 25% H_2_O, respectively. Although there was an enhancement (6.3%) on the conversion for the test of 15% H_2_O compared to without H_2_O, it is less than the others. The decrease observed in the conversion by increasing the water vapor from 10% to 15% for the test conducted at 800 °C cannot be easily explained and may be due to experimental error. When the temperature increased to 835 °C (Figure 1b), the positive effect of H_2_O on conversion can still be seen except in the case of 25% H_2_O. The conversion after 28 min of sulfation was 79.5% (Table 3) without H_2_O at 835 °C. With the introduction of water vapor, 6.8% and 2.5% enhancements were seen for 10% and 15% H_2_O, respectively. While the conversion after 28 min of sulfation was around 80% for the tests conducted at 835 °C with 0%, 10% and 15% H_2_O, it unexpectedly dropped down to 38% for 25% H_2_O. There is also no apparent positive impact of H_2_O on the total conversion at 870 °C (c).

Compared to the tests where water vapor was not used, the enhancement in the conversion after 28 min of sulfation at 800 °C was as high as 34% for the gas mixture containing 25% H_2_O. It was also around 8% for the gas mixture containing 10% H_2_O in tests conducted at 835 °C. At 870 °C, no enhancement was observed with the addition of water vapor into the gas mixture, and even in the test without water vapor the conversion was higher than 85%. When water vapor was added to the gas mixture at all three temperatures, it was observed that SO_2_ capture efficiency of limestone increased. However, as the TGA temperature increased (as the temperature approached the decomposition temperature of CaCO_3_), it was seen that the effect of the water vapor decreased. This clearly indicates that the water vapor content of the flue gas must always be taken into account for the direct sulfation of limestone operating the combustion systems at oxy-fuel conditions [17,22,24].

The TGA tests were repeated using the same amount of limestone and the same gas mixtures but at higher TGA temperatures. The tests were conducted at three different temperatures, 890 °C, 920 °C and 950 °C, to investigate the effect of water vapor on indirect sulfation of limestone at high temperatures during simulated oxy-fuel combustion conditions. While the total conversion was 27% without H_2_O at 890 °C (Figure 1d, Table 3), it reached up to 43% by introducing 10% H_2_O. Further increase of H_2_O share in gas mixture improved the total conversion and it increased to more than 70% for 20% and 25% of H_2_O. While the conversion after 28 min of sulfation was between 25% and 40% at 890 °C with H_2_O less than 15%, and it went up to 66% when more H_2_O was introduced at the same temperature (Table 3). The effect of water vapor on sulfation was less apparent at higher temperatures (920 °C (Figure 1e), and 950 °C (Figure 1f)). However, the conversion rose to 60%–80% range with the increase of temperature even in the absence of H_2_O.

**Figure 2:**
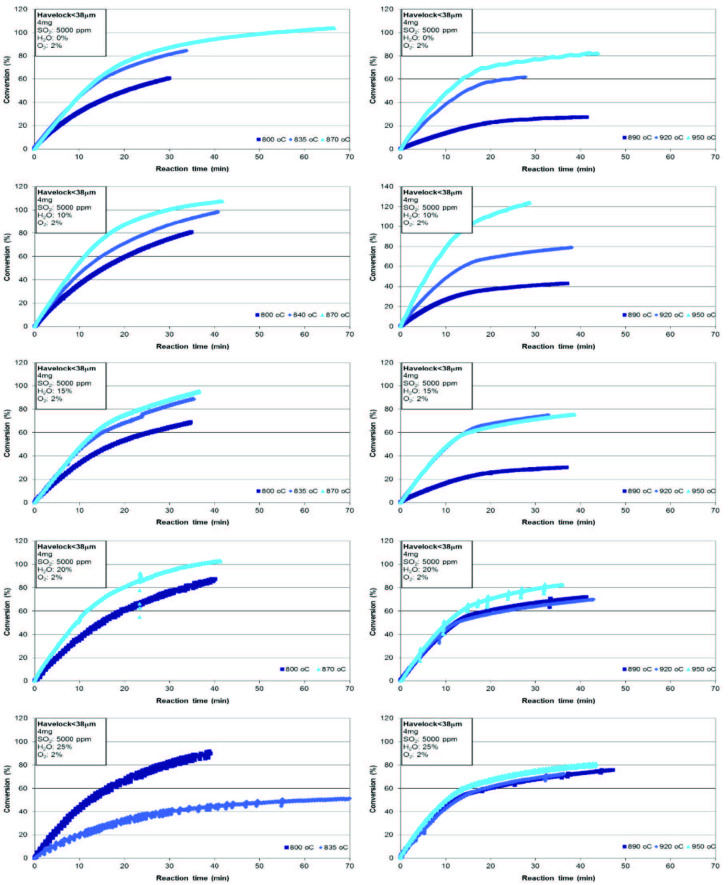
Effect of temperature on sulfation of Havelock limestone

In the case of lower three temperatures, 800 °C, 835 °C, and 870 °C, conversion of limestone improved with increasing temperature for the case of no H_2_O as can be seen in Figure 2. The same trend was also determined in the study of Wang et al. [17]. While total conversion was about 60% at 800 °C, it went up to 85% at 835 °C. The same positive effect on conversion was also seen for the case of 10%, 15%, and 20% H_2_O. Moreover, the addition of H_2_O into the gas mixture caused at least a 10% improvement on the conversion of limestone. These results show that there is a positive effect of H_2_O on sulfation of Havelock limestone. A similar trend was also reported during the sulfation of limestones under the synthetic flue gases contained 10% H_2_O and 15% CO_2_ [10]. The positive effect of H_2_O on sulfation of limestone at lower temperatures has been hypothesized as due to the transient formation of Ca(OH)_2_ as an intermediate product, which reacts with SO_2_ at a faster rate that CaCO_3_ does [17]. It has been observed that an increase on temperature had a negative effect on the conversion of limestone after 28 min of sulfation for the case of 25% H_2_O. While it was around 80% at 800 °C, it dropped down to 38% at 835 °C (Table 3). However, the decrease in conversion could not be explained. One possible explanation is that there may be an interference between sulfation and calcination at higher temperatures. Sulfation becomes faster at higher temperatures, but the pores of CaCO_3_ may be blocked by the sulfate and the actual calcination be drastically slowed. As the sulfation becomes faster as the temperature is increased, a reverse impeding effect can slow down calcination [16].

Conversion of limestone for higher temperatures (890 °C, 920 °C, and 950 °C) as a function of time can be seen in Figure 2. It was observed that total conversion of limestone increased with temperature for the cases of 0%, 10%, and 15% H_2_O. For the tests conducted at 20% and 25% H_2_O, the total conversion did not change greatly at different temperatures and was between 70% and 80%.

The studies conducted at a high CO_2_ concentration (representing oxy-fuel combustion conditions) in a TGA or a tube furnace are presented in Table 4 for the comparison purposes. Wang et al. [17] investigated the effect of water vapor (10%) on the conversion of limestone at temperatures of 800 and 850 °C for 3 different limestones in a TGA. The gas mixture fed to the TGA was composed of 80% CO_2_, 4% O_2_, and 5000 ppm_v_ SO_2_. They carried out their experiments without water vapor and by adding 10% water vapor to the gas mixture. Nitrogen gas was used as a balance gas. For all 3 limestones, adding water vapor to the gas mixture at both temperatures resulted in a better conversion. When the results of the study are compared with the results of this study, it is seen that higher conversion values were obtained in this study (Table 3; 61% and 81%, respectively for 0% and 10% H_2_O at 800 °C, cycles for 0% and 10% H_2_O at 835 °C, 85% and 98%, respectively) at temperatures lower than 850 °C. This difference may be due to the different particle size used. Wang et al. [17] studied the particle size of 75–125 µm and it is generally accepted that conversion decreases as the particle size increases [17]. Stewart et al. [22] also obtained higher conversion values in their TGA tests conducted with Cadomin limestone at 850 °C. It was reported that the conversion increased with the amount of water vapor in the gas mixture. However, the maximum amount of water vapor in the gas mixture was 15% in that study. Duan et al. [24] increased the percentage of water vapor in the gas mixture up to 40%. They carried out sulfation tests in a tube furnace at temperatures of 800 °C and 850 °C. Two types of limestone (American and Chinese) were used, and it was observed that the water vapor enhanced the conversion. These studies investigating direct sulfation mechanisms for limestone were carried out under oxy-fuel combustion conditions at low temperatures (≤850 °C). While García-Labiano et al. [27] conducted tests at high temperatures (≥900 °C), unfortunately, they did not study the effect of water vapor on sulfation.

**Table 4:** tudies conducted in a TGA or a tube furnace at oxy-fuel conditions.

	Limestone	T	CO_2_	H_2_O	O_2_	SO_2_	Conversion
		°C	%	%	%	%	%
[17]	Kelly Rock	800	80	0/10	4	0.5	39/43
[17]	Kelly Rock	850	80	0/10	4	0.5	28/57
[17]	Havelock	850	80	0/10	4	0.5	28/51
[17]	Calpo	850	80	0/10	4	0.5	35/67
[22]	Cadomin	850	balance	0/7.5/15	2.53	0.38	80/87/96
[24]	American	800	balance	40	7	3	72
[24]	American	850	balance	0/10/20/30/40	7	3	70/73/75/80/82
[24]	Chinese	800	balance	40	7	3	65
[24]	Chinese	850	balance	0/10/20/30/40	7	3	67/88/90/92/93
[27]	Granicarb	800	80			0.3	40
[27]	Granicarb	850	80			0.3	73
[27]	Granicarb	900	80			0.3	79

**Figure 3:**
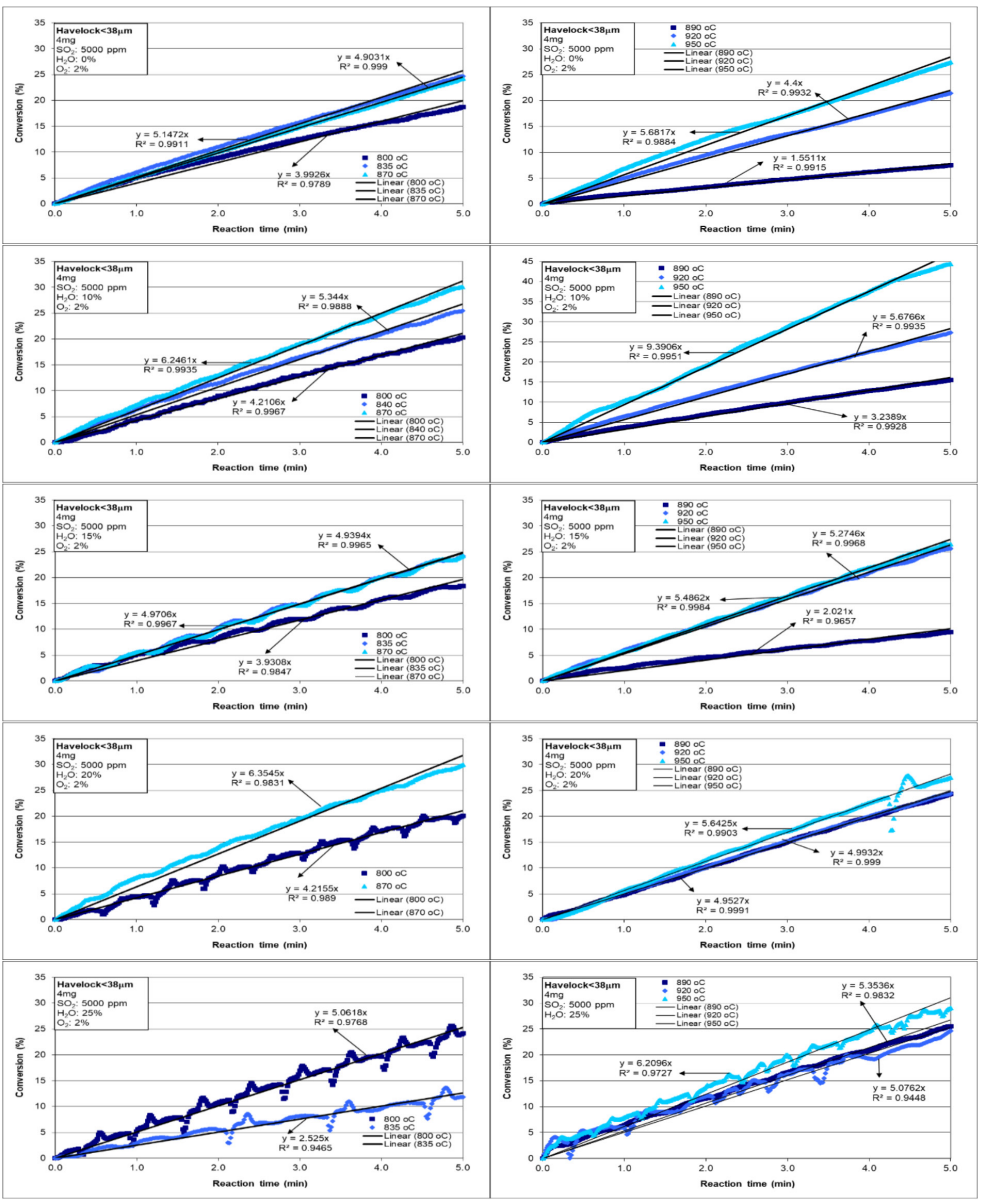
Effect of temperature on sulfation of Havelock limestone within the first 5 min.

In order to examine the intrinsic reaction kinetics, it is more important to focus on the conversion rate in the first five minutes than to look at total conversion. For that purpose, the graphs in Figure 3 were drawn. The conversion within the first 5 min after sulfation started is shown in Figure 3. For each case, trendlines according to the best R2 were drawn and the slopes of these lines were determined. When the conversion values of Havelock limestone after 28 min of sulfation in were examined, it can be seen that the conversion at temperatures (800 °C, 835 °C, and 870 °C) for direct sulfation was higher than at temperatures (890 °C, 920 °C, and 950 °C) for indirect sulfation as a general trend. When the slopes of each line in Table 3 were examined in detail, as expected it was seen that Havelock limestone at higher temperatures sulfated at a faster rate than at lower temperatures. Indirect sulfation allowed higher conversion rates for the first five minutes than direct sulfation. However, in general, the conversion of Havelock limestone for direct sulfation was greater than for indirect sulfation.

**Figure 4:**
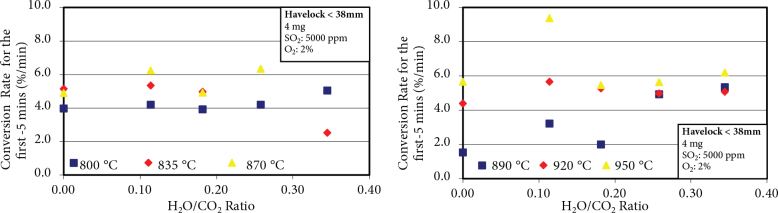
Conversion rate as a function of H_2_O/CO_2_ ratio.

Conversion rates determined from the slopes of each line for the first five minutes in Figure 3 were plotted in Figure 4 as a function of H_2_O/CO_2_ ratio. At 800 °C, it was observed that conversion rate increased with H_2_O/CO_2_ ratio, except for the H_2_O/CO_2_ ratio of 0.18. While the conversion rate was 4.0%/min for no H_2_O, it increased to 5.1%/min when H_2_O/CO_2_ ratio was 0.34. Conversion rates did not change much for H_2_O/CO_2_ ratio of 0–0.2 at 835 °C. However, when H_2_O/CO_2_ ratio exceeded 0.2, there was a rapid decrease in the conversion rate. The results for the conversion rates at 870 °C, do not permit us to say whether there is a positive or negative impact of water on sulfation.

However, there was a positive impact of water on conversion rates at 890 °C. While the conversion rate without water at 890 °C was 1.6%/min, it increased with the addition of water in the gas mixture and went up to 5.4%/min for 25% H_2_O. There was one outlier rate, which was for H_2_O/CO_2_ ratio of 0.18 at 890 °C. This point in Figure 3 seems to be inconsistent with the general trend observed. At 920 °C, the conversion rate increased until H_2_O/CO_2_ ratio was 0.15, and in cases where H_2_O/CO_2_ ratio was greater than 0.15, it showed a decreasing trend. If the conversion rate at H_2_O/CO_2_ ratio of 0.11 is excluded, possibly because there is some other factor intervening, it can be concluded that H_2_O/CO_2_ ratio does not have a significant effect on the conversion rate at the highest temperature of our study, namely 950 °C.

The H_2_O in gas mixture at 800 °C improved the conversion of limestone during the sulfation process. When the temperature increased up to 835 °C, the positive effect of H_2_O on conversion could be still seen except in the case of 25% H_2_O. In this case, conversion unexpectedly dropped down to 50% from 90% levels. The positive impact of H_2_O on conversion was also seen at 870 °C. When water vapor was added to the gas mixture at lower temperatures (800 °C, 835 °C, and 870 °C), it was observed that SO_2_ capture efficiency of limestone also increased. However, as the temperature increased, it was seen that the enhancement in total conversion values decreased. In the case of the lower three temperatures, 800 °C, 835 °C, and 870 °C, conversion of limestone improved with increasing temperature for the case of no H_2_O present. While total conversion was about 60% at 800 °C, it went up to 85% at 835 °C. The same positive effect on conversion was also seen for the case of 10%, 15%, and 20% H_2_O. Moreover, addition of H_2_O caused at least 10% improvement on the conversion of limestone. Havelock limestone at higher temperatures (890 °C, 920 °C, and 950 °C) achieved a greater conversion rate than at lower temperatures (800 °C, 835 °C, and 870 °C) for direct sulfation in the first five minutes. However, total conversion of Havelock limestone for direct sulfation was greater than for indirect sulfation as a general trend. This clearly indicates that the water vapor content of the flue gas should be always taken into account for the sulfation of limestone under oxy-fuel conditions.
